# Long‐term outcomes in a 25‐year‐old female affected with lipin‐1 deficiency

**DOI:** 10.1002/jmd2.12016

**Published:** 2019-03-14

**Authors:** Karolina M. Stepien, Wolfgang M. Schmidt, Reginald E. Bittner, Orna O'Toole, Brian McNamara, Eileen P. Treacy

**Affiliations:** ^1^ Mark Holland Metabolic Unit, Adult Inherited Metabolic Diseases Salford Royal NHS Foundation Trust Salford United Kingdom; ^2^ Neuromuscular Research Department Center for Anatomy and Cell Biology, Medical University of Vienna Vienna Austria; ^3^ Department of Neurology Mercy University Hospital Cork Ireland; ^4^ Department of Clinical Neurophysiology Cork University Hospital Cork Ireland; ^5^ University College Dublin Dublin Ireland; ^6^ Paediatrics Department Trinity College Dublin Ireland; ^7^ National Centre for Inherited Metabolic Diseases The Mater Misericordiae University Hospital Dublin Ireland

**Keywords:** lipid myopathy, lipin‐1 deficiency, *LPIN1* gene, mutation, neuropathy, rhabdomyolysis

## Abstract

Lipin‐1 is a phosphatidic acid phosphohydrolase (EC 3.1.3.4) that catalyzes the dephosphorylation of phosphatidic acid to diacylglycerol and inorganic phosphate. Deficiency of this enzyme causes potentially fatal severe, recurrent episodes of rhabdomyolysis triggered by infection. The defect has only recently been recognized so little is known about the long‐term outcome in adult patients with this disorder. We report the course and outcome of a 25‐year‐old female patient with lipin‐1 deficiency after a recent episode of rhabdomyolysis requiring intensive care admission with a peak creatine kinase of 500 000 IU/L. One‐year post discharge from intensive care, the patient has residual drop foot bilaterally consistent with bilateral common peroneal neuropathies in addition to a background residual distal myopathy.

## 1 INTRODUCTION

Lipin‐1 deficiency is one of the genetic causes of rhabdomyolysis. It affects patients of various ethnic backgrounds^1^ and often before the age of 6 with life‐threatening myoglobinuria crises.^2^, ^3^, ^4^, ^5^, ^6^ The massive rhabdomyolysis episodes in humans are often precipitated by febrile illness,^4^ heat,^7^ fasting, and anesthesia.^2^, ^3^, ^8^

The *LPIN1* deleterious mutations lead to stop codons or internal deletions.^4^, ^9^, ^10^ Heterozygous *LPIN1* mutations have been identified in two individuals with statin‐induced myopathy, suggesting that partial lipin‐1 deficiency might represent a risk factor for drug‐induced myotoxicity and exercise‐induced myalgia.^4^, ^9^

The *LPIN1* gene belongs to the *LPIN* gene family (which includes three isoforms). The lipin‐1 protein, the muscle‐specific phosphatidic acid phosphatase 1 (PAP1) (EC 3.1.3.4), is a key enzyme that catalyzes the conversion of phosphatidic acid (phosphatidate) to diacylglycerol in the triacylglycerol (TAG) synthesis pathway.^5^, ^11^, ^12^, ^13^, ^14^, ^15^ Deficiency of this enzyme causes accumulation of phosphatidic acid and lysophospholipids in muscle tissue and recurrent rhabdomyolysis.^3^

Lipin‐1 is an 890 amino acid intracellular protein^3^ most abundant in adipocytes, skeletal muscle fibers, liver, and myocardium,^13^, ^16^ and is subject to regulation by phosphorylation/dephosphorylation by mTOR.^4^

## 2 CASE

A 25‐year‐old female Irish patient presented with recurrent episodes of rhabdomyolysis since childhood. The first episode occurred at the age of 22 months following a respiratory tract infection. At that time, her creatine kinase (CK) serum concentration was noted to be 250 000 U/L.

The family history indicated that both parents were well. However, two siblings (brother and sister) had sudden death at the age of 2 and 4 years more than 20 years ago following a short infectious illness with sudden deterioration over a period of hours. In both cases, the children became progressively weak with severe muscle pain and had evidence of rhabdomyolysis and myoglobinuria of uncertain etiology. Skeletal muscle histology and electron microscopy studies at postmortem evaluation were normal in both children, cardiac evaluation demonstrated dilated cardiomyopathy. Notes pertaining to their clinical episodes were not available. On first presentation, our patient had no hypoglycemia and no ketosis during the acute illness, but was initially treated as a possible long chain fatty acid oxidation defect due to the family history.

The patient had numerous subsequent admissions with similar presentations of extremely elevated CK concentration (1 000 000 U/L at the age of 9) associated with muscle pain. This was despite aggressive carbohydrate supplementation including nocturnal cornstarch. Her fat intake from food was continually restricted to approximately 40 g/d. Her total fat intake was supplemented with the use of MCT Oil (medium chain triglycerides supplement) and essential fatty acids in the form of walnut oil. Coenzyme Q_10_ at a dose of 100‐200 mg daily was provided on an ongoing basis. The CK concentration was also raised between the episodes (500‐2000 U/L). She was advised to limit her exercise to 20 minutes per day and have high‐calorie drink prior to any physical activity.

Extensive investigations were performed over the presenting years with the lack of a definite etiology. Serial urine organic acid profile and the acylcarnitine profiles were normal. A fibroblast fatty acid oxidation study showed normal myristate and palmitate oxidation studies in fibroblasts. Genetic analysis for fatty acid oxidation defect (LCHAD, MCAD, CPT I, and CPT II) and McArdle disease was uninformative.

**Figure 1:**
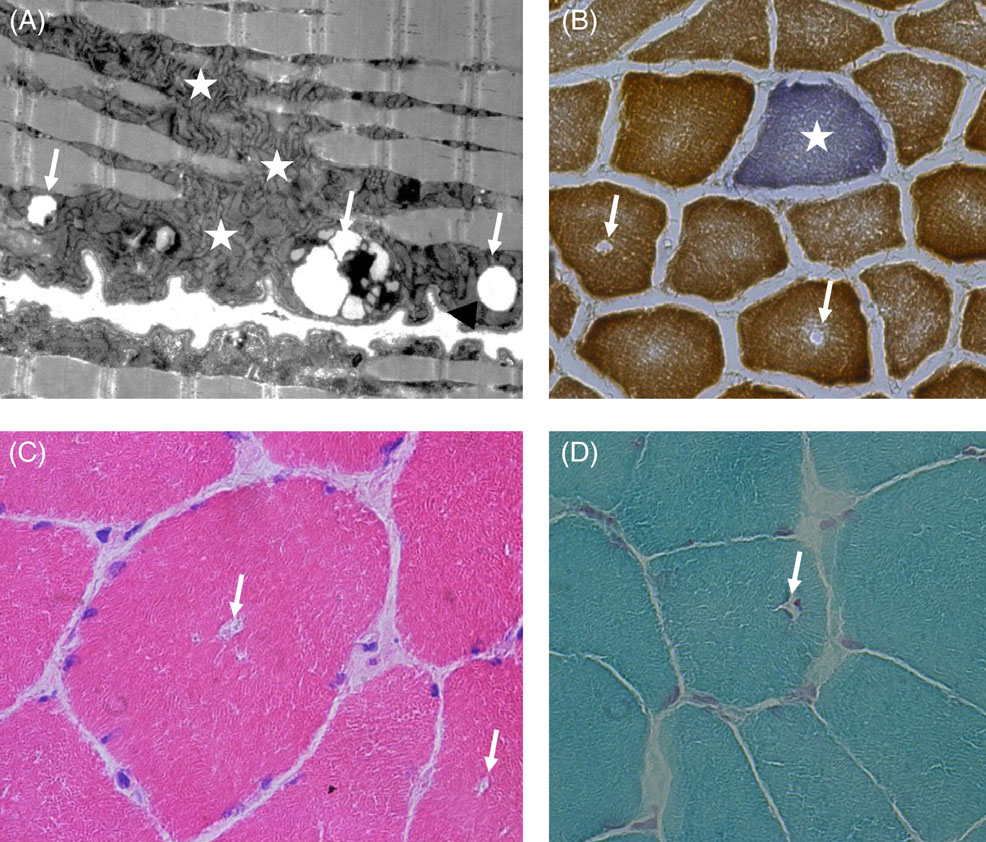
Muscle biopsy. A, Electron micrograph showing numerous intramyocellular lipid (IMCL) (arrows) as well as aggregates of malformed mitochondria (asterisks). The middle arrow is not a classical IMCL but likely mitochondria and lipids undergoing mitophagy. B, A single cytochrome‐c‐oxidase‐negative muscle fiber. C, Rimmed vacuoles (arrows, H&E). D, Rimmed vacuole (arrow, Gomori Trichrome)

At the age of 16 years, mitochondrial respiratory chain activity measured in a muscle biopsy was normal, however morphological findings, such as intramyocellular lipid, were compatible with lipin‐1 deficiency (Figure 1). Her echocardiogram and electrocardiogram did not show any abnormal findings.

At the age of 19 years, DNA sequence analysis of the *LPIN1* gene (all coding exons and flanking intron boundaries corresponding to the canonical transcript variant NM_145693.3) revealed the presence of a common pathogenic intragenic deletion within the *LPIN1* gene (c.2295‐866_2410‐30del1763) encompassing exon 18 (HGMD accession: CG085181). However, a second mutation could not be identified.

**Figure 2:**
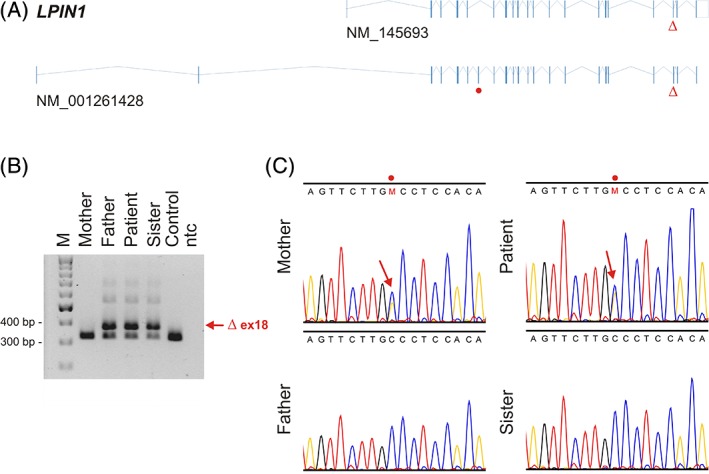
Genetic analysis of the *LPIN1* gene. A, Exon‐intron structure of *LPIN1* transcripts NM_145693 and NM_001261428. The nonsense mutation located in an alternative exon annotated in NM_001261428 only is indicated by a red dot. The common deletion comprising exon 18 and affecting both transcripts is indicated by the Δ symbol. B, Agarose gel electrophoresis loaded with reaction products of an assay probing for presence of the common deletion. The assay used a common forward primer in intron 17 (5’‐GGTCTGGCACATCTTCTGTTAGAT‐3′) in conjunction with two reverse primers, the first located within the deleted region (5’‐CTGTATTGCCTGTCCTATCAAATG‐3′) giving rise to a 326 bp product, and the second located downstream of the deleted region within exon 19 (5’‐ACTCACGAAGAGATGTTGGTCTTT‐3′), giving rise to 379 bp PCR‐product in case the deletion is present (indicated by an “Δ ex18”). “M”: 100 bp molecular weight marker (the 300 and 400 bp bands are indicated on the left), “Control”: DNA from a healthy unrelated individual, “ntc”: no template PCR control. The longer weak bands correspond to heteroduplices of both PCR products. The patient, her father, and her unaffected sister are heterozygous carriers of the common deletion. C, Capillary DNA sequencing in the patient's family revealing a heterozygous point mutation within the alternative exon annotated in NM_001261428 only (indicated by an arrow within the trace and marked by a red dot above the sequence) in the patient and her mother. Thus, the patient is compound heterozygous for the deletion and the point mutation

Subsequently, the *LPIN1* coding region was analyzed by reverse‐transcriptase PCR (RT‐PCR) from total RNA isolated from muscle tissue and conventional DNA sequencing (Figure S1A,B). In addition to transcripts lacking exon 18 or exons 18‐19 (corresponding to the allele harboring the genomic exon 18 deletion), an alternative exon spliced in between exon 5 and exon 6 was detected in a significant proportion of transcripts (Figure S1C,D). Because this alternative exon, named exon 5a, corresponded to an alternatively spliced in‐frame exon annotated only in an N‐terminal *LPIN1* transcript variant (NM_001261428.2), further targeted DNA‐based sequencing was performed. Indeed, this identified a second heterozygous variant (NC_000002.11:g.11916284C>A), which was formally regarded a nonsense mutation introducing a premature stop‐codon within exon 5a (which would correspond to NM_001261428.2:c.942C>A, NP_001248357.1:p.[Cys314*]; Figure 2). RT‐PCR of total RNA isolated from healthy skeletal muscle detected exon 5a also in transcripts containing the first (noncoding) exon of the canonical isoform (*data not shown*). However, because exon 5a is currently not annotated to be contained in this main reference transcript (NM_145693.3), further studies will be needed to clearly establish pathogenicity of the novel variant.

Segregation analysis in the patient's family revealed that her father and younger sister are heterozygous for the common pathogenic intragenic deletion (NM_145693.3:c.2295‐866_2410‐30del1763), while her mother was a heterozygous carrier of the variant within the alternative exon. Thus, the results of these molecular genetic analyses were formally consistent with the clinical diagnosis of *LPIN1*‐associated rhabdomyolysis, due to compound heterozygosity for a known pathogenic deletion and potentially pathogenic novel mutation.

### 2.1 Recent admission

At the age of 25 years, the patient presented with acute muscle pain and weakness following prolonged fasting and strenuous exercise. A rhabdomyolysis crisis was confirmed with a CK of 500 000 U/L. She was admitted to intensive care unit (ICU) for a 2 week period over which period she lost a significant amount of weight. During her critical illness, she received an intravenous infusion of 10% dextrose at 1.5 times maintenance with added electrolytes, sodium bicarbonate, morphine, and dexamethasone. Intravenous carnitine was also provided as the patient had previously observed this to be clinically beneficial. She was treated symptomatically, with shortened fasting periods, corn‐starch at night and an “unwell dietary regimes” with a mild restriction of fat (40 g per day) and supplementation with MCT oil and walnut oil for essential fatty acids and Liquigen 5 g daily. On day 11, her CK was monitored 4 hourly and was 1248 U/L. On discharge from ICU, this patient had generalized muscle weakness, stiff lower limb muscles (gastrocnemius), and bilateral drop foot requiring orthotic splints. Muscle weakness gradually improved after months of rehabilitative physiotherapy. Her drop foot has improved somewhat (power from 0/5 to 3/5 for dorsiflexion and extensor hallicis longus over 1 year) with areas of altered *sensation* anterolaterally below the knees consistent with bilateral residual common peroneal neuropathies (CPN). Serial nerve conduction/electromyography studies demonstrated bilateral CPN palsies and a background generalized myopathy. The most likely cause of this patient's weakness was a critical care neuromyopathy, which has improved with time, in addition to CPN palsies related to significant weight loss while critically ill. However, we cannot exclude the possibility that both defects were related to her lipin‐1 deficiency. While myopathy has been reported in a few cases, CPN damage is not a known association of this rare metabolic disorder.

## 3 DISCUSSION

Despite the well‐known roles of lipin‐1 in lipid biosynthesis and transcriptional regulation, the pathogenic mechanisms leading to rhabdomyolysis remain unknown. Lipin‐1 of the lipin family accounts for the majority of PAP activity in human and mouse skeletal muscle,^10^ and the transgenic modulation of muscle lipin‐1 activity alters energy balance.^13^ Metabolic stress due to impairment of mitochondrial functions and autophagy was previously shown to lead to skeletal muscle myofibrillar necrosis.^16^ Therefore, it was hypothesized that damaged mitochondria, energy producing organelle, might accumulate in lipin‐1‐deficient muscle because of impaired autophagy.^6^ Lipin‐1 appears to be the predominant lipin‐1 in the heart because of the apparent absence of cardiac PAP activity in lipin‐1‐deficient cases.^13^ In our patient, however, the myocardium was not affected and echocardiogram was reported as normal.

The phenotype might be explained by the nearly ubiquitous expression of two other *LPIN* genes, *LPIN2* and *LPIN3*, encoding closely related proteins.^13^ Lipin‐1, lipin‐2, and lipin‐3 share homologous sequences and overlapping PAP1 enzymatic functions.^17^ Lipin‐2 and lipin‐3 can potentially compensate for the absence of lipin‐1.^13^, ^17^ Michot et al.^4^ showed that primary myoblasts from lipin‐1‐deficient patients exhibit a dramatic decrease in *LPIN1* expression and PAP 1 activity, and a significant accumulation of lipid droplets presents associated with *LPIN1*‐dependent rhabdomyolysis as a novel form of lipid myopathy.^6^, ^9^, ^10^ Pelosi et al.^16^ further demonstrated that accumulation of neutral lipids is determined by a balance among lipid biosynthesis, lipid hydrolysis, and β‐oxidation^16^ but it remains unclear how this relates to the massive muscle breakdown in affected patients.^6^

It is possible that muscle‐specific mechanisms compensate for lipin‐1 deficiency or that the transcriptional function of lipin‐1 is less important for the expression of the genes in the muscle cells.^4^ Furthermore, the absence of obvious metabolic and the limited transcriptional consequences of lipin‐1 deficiency in normal culture conditions supports the apparent lack of symptoms between rhabdomyolysis episodes.^4^ It also implies that mitochondrial function is adequate to meet energy demands during everyday activities.

Another feature of lipin‐1‐related myopathy noted is the presence of endomysial inflammatory infiltrates, mostly composed of CD8 and CD4 lymphocytes. As shown by a muscle biopsy, there are residual granular microcalcifications and terminal complement complex deposits evident compatible with necrotic myopathy and early rhabdomyolysis.^18^ Several studies have also demonstrated that lipin‐1 has potent anti‐inflammatory properties.^19^ Lipin‐1 is a major contributor to macrophage proinflammatory activation.^19^ Pro‐inflammatory stress induced by TNFα + IL‐1β or poly(I:C), a synthetic inducer of the antiviral response, exacerbated lipid deposits accumulation, has been shown to decrease carnitine palmitoyltransferase I (CPT1) activity and increase TAG content, highlighting a crucial role of inflammation in the pathogenesis of lipin‐1 deficiency. The fact that in humans episodes of myoglobinuria are mostly precipitated by febrile illnesses^4^ emphasizes a critical role of inflammatory stress response as a potential triggering factor of rhabdomyolysis.^4^ In addition, prolonged exercise and excess of circulating‐free fatty acids also induce a mild state of inflammation. In these situations, the muscles secrete various cytokines and chemokines, which may amplify immune response in a paracrine/autocrine fashion.^20^, ^21^ As it has also been shown that prolonged exercise may induce inflammation, we advised our patient to reduce the frequency and intensity after the severe rhabdomyolysis.

### 3.1 Long‐term management of Lipin‐1 deficiency

Current rhabdomyolysis guidelines emphasize that with regard to the symptomatic treatment of rhabdomyolysis in lipin‐1 deficiency, early detection, aggressive hydration, high energy intake from carbohydrates, and monitoring for hyperkalemia and cardiac arrhythmias are important.^8^

To avoid long‐term complications, prevention of fever, heat, and excessive exercise, as well as prompt treatment of acute illness is essential. High carbohydrate supplement drinks as well as high carbohydrate meals may be helpful in calorie provision when oral intake is possible. In our case, lipids contributed less than 30% of total energy intake during the acute illness. Oral l‐carnitine provided at the dose of 1000 mg taken three times a day may have been effective in regaining strength in the lower limbs subsequent to the last rhabdomyolysis attack. l‐carnitine may also be administered intravenously during an acute presentation.^8^

In view of the hypothesis that synergistic interaction among lipid metabolism, lipid signaling, and inflammation^8^ may underlie the severe rhabdomyolysis crises of lipin‐1 deficiency, new treatment possibilities come to light. Dexamethasone was shown to decrease the number of lipid droplets in lipin‐1 deficient patients' myoblasts with a decrease in peak CK concentration during acute rhabdomyolysis. This therapy may prove to be beneficial for severe decompensation.^8^

### 3.2 Lipin‐1 deficiency and neuropathy

A study by Nadra et al.^22^ showed that the lack of PAP activity in Schwann cells in lipin‐1 deficient mice was sufficient to cause peripheral neuropathy associated with myelin degradation and attenuated nerve conduction velocity.^22^ So far however, peripheral neuropathy has not been described in a human being affected with this genetic defect.

Peripheral neuropathy is a recognized complication of acute illness whereby severe nerve damage may result from immobilization, rhabdomyolysis, tissue necrosis, and compartment syndrome. Symmetrical peripheral neuropathy is often observed in systemic disease. In our case, nerve conduction study performed shortly after the acute presentation to ICU showed bilateral CPN affecting the left side more than the right, most likely due to compression at the knee. Her acute weight loss during the acute illness could also have contributed to the CPN. Acute on chronic myopathy in this case was most likely consistent with critical care myopathy in the setting of lipin‐1 myopathy. However, as the left deep peroneal motor and superficial peroneal sensory response were unilaterally absent, and on the right‐hand side a superficial peroneal sensory response was present, it was less likely to be a generalized motor‐sensory neuropathy.

Eight months later, a repeat nerve conduction study confirmed myopathy and additional bilateral CPNs, the left side more affected than the right. There was evidence of chronic myopathy suggesting that the critical care element has improved but she may have a residual lipin‐1‐related myopathy and the CPN which continued. It was uncertain whether it would ever improve. To the best of our knowledge, our patient was not known to have an established peripheral neuropathy prior to this acute presentation.

### 3.3 *LPIN1* gene mutations

Over 27 *LPIN1* gene mutations have been described to date in several ethnic groups but no clear genotype‐phenotype correlation has been shown to date.^8^ The canonical *LPIN1* transcript isoform contains 20 exons (of which 19 are coding) and a deletion mutation spanning exon 18 is noted in 86% of Caucasian patients.^8^

Nearly all *LPIN1* mutations that cause childhood rhabdomyolysis are nonsense or deletion mutations, predicted to result in an inactive protein.^4^, ^5^, ^9^ Myopathy has also been reported in individuals that are heterozygous for *LPIN1* missense mutations in response to statin drug treatment.^5^, ^6^, ^9^

To date, all reported *LPIN1* mutations affect the isoform corresponding to reference NM_145693.3 (which is also used in the HGMD). This is the first patient with a *LPIN1* mutation affecting a non‐canonical exon that is contained in three currently known isoforms, of which only one is well‐supported: NM_001261428.2. The latter isoform is the longest, coding for a 975 amino acid‐long protein variant called isoform 3 (NP_001248357.1). Our data suggest that transcripts with and without this alternative exon are expressed also in healthy muscle tissue (Figure S1D), prompting its inclusion in targeted sequencing in the diagnostic setting. However, a clear limitation of the present report is that definitive pathogenicity of the point mutation could not be established due to lack of experimental evidence on the protein level. Future studies are therefore warranted to clarify the significance of exon 5a in *LPIN1*‐associated rhabdomyolysis.

## 4 CONCLUSION

We present the case of a patient with lipin‐1 deficiency now aged 25 years. The patient has had multiple episodes of rhabdomyolysis from which she has recovered reasonably well. After her most recent attack, she has been left with residual drop foot bilaterally due to CPN with incomplete improvement over a 1‐year period. Electrically, she also has a lower limb myopathy from which she is asymptomatic. CPN has not previously been reported in lipin‐1 deficiency although etiology in this case may be related to severe weight loss and critical illness.

## CONFLICTS OF INTEREST

The authors have no conflicts of interest to report.

## AUTHOR CONTRIBUTIONS

All authors read and approved the manuscript before submission.

*Karolina M. Stepien*: Conception and design, analysis and interpretation of data, drafting the chapter, revising the chapter critically for important intellectual content.

*Wolfgang M. Schmidt*: Conception and design, analysis and interpretation of data, drafting the chapter, revising the chapter critically for important intellectual content; data interpretation.

*Reginald E. Bittner*: Conception and design, analysis and interpretation of data, drafting the chapter, revising the chapter critically for important intellectual content; data interpretation.

*Orna O'Toole*: Conception and design, analysis and interpretation of data, drafting the chapter, revising the chapter critically for important intellectual content.

*Brian McNamara*: Conception and design, analysis and interpretation of data, drafting the chapter, revising the chapter critically for important intellectual content.

*Eileen P. Treacy*: Conception and design, analysis and interpretation of data, drafting the chapter, revising the chapter critically for important intellectual content.

## PATIENT CONSENT

Patient's consent was obtained.

## Supporting information

Figure S1 RNA‐based analysis of the *LPIN1* gene (RT‐PCR). A. Exon‐intron structure of the canonical *LPIN1* transcript annotated with: location of specific cDNA primer (filled arrowhead, 5’‐CTTCCTATCTTGCTTAGAAATGTCAGC‐3′), and PCR primers for amplifying a 5′‐part (PCR I, 1873 bp; exon 2 forward: 5’‐ATGAATTACGTGGGGCAGTTAG‐3′ and exon 14 reverse: 5’‐TGTAGCTGACATTAGGCAGAAGAG‐3′) and a 3′‐part (PCR II, 1305 bp; exon 10 forward: 5′‐ ATCTCGTGGTAAAGATTGGGAGTA‐3′ and 3’‐UTR reverse: 5’‐AAATGCTTCTCAATTCTCTCTGCT‐3′) of the complete *LPIN1* coding region (exons 2‐20) are indicated. Locations of primers used for sequencing across the critical exon 5: exon 6 junction of amplicon PCR I (reverse primer in exon 7, 5′‐ GACTCTTTCATCTTGTGTGGAGAA‐3′), and the exon 17: exon 18 junction in amplicon PCR II (forward primer in exon 16, 5’‐TGTACCATAAAGTGAGCCAGAATG‐3′), respectively, are indicated by open arrowheads. The nonsense mutation located in an alternative exon, which is in intron 5 of the canonical transcript but annotated as coding exon in transcript NM_001261428, is indicated by a red dot. The common genomic deletion of exon 18, leading to transcripts lacking either exon 18 or exon 18 and exon 19, is indicated by the Δ symbol. B. Agarose gel electrophoresis loaded with RT‐PCR reaction products of *LPIN1*‐cDNA prepared from total RNA isolated from the patient's muscle biopsy (patient) and a healthy control (control). “M”: 100 bp molecular weight marker (GeneRuler 100 bp Plus; bands in the range from 1 to 2 kb are indicated); “ntc”: no template PCR control. All PCR products (note the three distinct bands in the lane loaded with reaction product from patient's PCR II) were subjected to direct DNA sequencing without purification. C,D. Differential reading of mixed sequencing traces of products from PCR I revealing presence of the alternative exon spliced in between exon 5 and exon 6 (denoted “exon 5a”) in a significant proportion of transcripts in both, total RNA isolated from the patient's muscle tissue (C) and from a healthy control as well (D). Sequencing traces of products from PCR II revealed transcripts containing or lacking exons 18 or lacking exons 18‐19 in RNA isolated from the patient's muscle tissue (C), but not from a healthy control (D)
