# Supplementary figures and images for: Long‐term outcomes in a 25‐year‐old female affected with lipin‐1 deficiency
Source: JIMD Rep. 2019 Mar 14;46(1):4–10. doi: 10.1002/jmd2.12016 (PMC6498837; doi:10.1002/jmd2.12016)

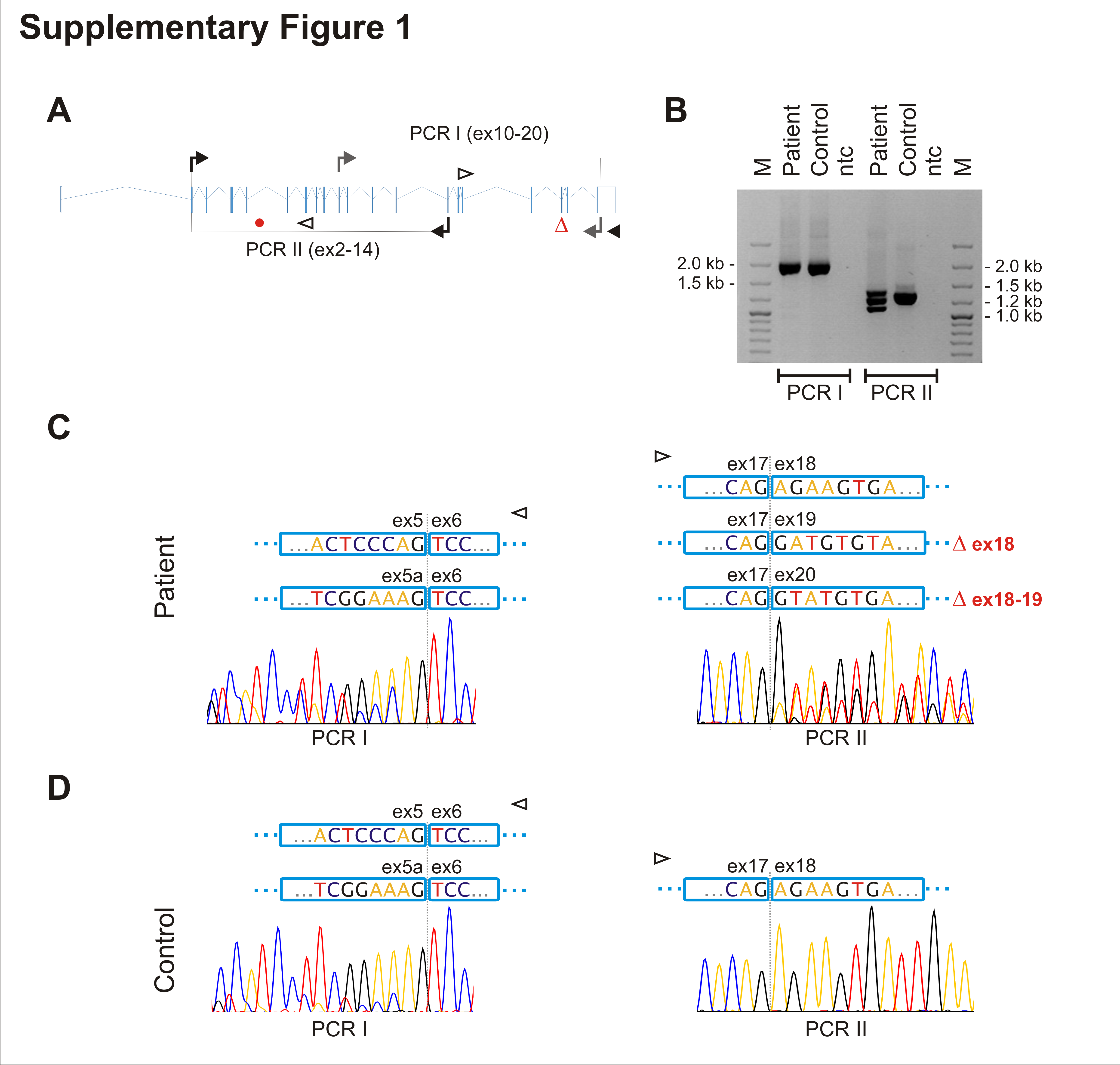

Supplement: Supplementary file 1 — Figure S1 RNA‐based analysis of the LPIN1 gene (RT‐PCR). A. Exon‐intron structure of the canonical LPIN1 transcript annotated with: location of specific cDNA primer (filled arrowhead, 5’‐CTTCCTATCTTGCTTAGAAATGTCAGC‐3′), and PCR primers for amplifying a 5′‐part (PCR I, 1873 bp; exon 2 forward: 5’‐ATGAATTACGTGGGGCAGTTAG‐3′ and exon 14 reverse: 5’‐TGTAGCTGACATTAGGCAGAAGAG‐3′) and a 3′‐part (PCR II, 1305 bp; exon 10 forward: 5′‐ ATCTCGTGGTAAAGATTGGGAGTA‐3′ and 3’‐UTR reverse: 5’‐AAATGCTTCTCAATTCTCTCTGCT‐3′) of the complete LPIN1 coding region (exons 2‐20) are indicated. Locations of primers used for sequencing across the critical exon 5: exon 6 junction of amplicon PCR I (reverse primer in exon 7, 5′‐ GACTCTTTCATCTTGTGTGGAGAA‐3′), and the exon 17: exon 18 junction in amplicon PCR II (forward primer in exon 16, 5’‐TGTACCATAAAGTGAGCCAGAATG‐3′), respectively, are indicated by open arrowheads. The nonsense mutation located in an alternative exon, which is in intron 5 of the canonical transcript but annotated as coding exon in transcript NM_001261428, is indicated by a red dot. The common genomic deletion of exon 18, leading to transcripts lacking either exon 18 or exon 18 and exon 19, is indicated by the Δ symbol. B. Agarose gel electrophoresis loaded with RT‐PCR reaction products of LPIN1‐cDNA prepared from total RNA isolated from the patient's muscle biopsy (patient) and a healthy control (control). “M”: 100 bp molecular weight marker (GeneRuler 100 bp Plus; bands in the range from 1 to 2 kb are indicated); “ntc”: no template PCR control. All PCR products (note the three distinct bands in the lane loaded with reaction product from patient's PCR II) were subjected to direct DNA sequencing without purification. C,D. Differential reading of mixed sequencing traces of products from PCR I revealing presence of the alternative exon spliced in between exon 5 and exon 6 (denoted “exon 5a”) in a significant proportion of transcripts in both, total RNA isolated from the patient's m [file JMD2-46-4-s001.png]
